# Missed Opportunities for HIV Testing and Late-Stage Diagnosis among HIV-Infected Patients in Uganda

**DOI:** 10.1371/journal.pone.0021794

**Published:** 2011-07-05

**Authors:** Rhoda K. Wanyenze, Moses R. Kamya, Robin Fatch, Harriet Mayanja-Kizza, Steven Baveewo, Sharif Sawires, David R. Bangsberg, Thomas Coates, Judith A. Hahn

**Affiliations:** 1 Department of Disease Control and Environmental Health, Makerere University School of Public Health, Kampala, Uganda; 2 Department of Medicine, Makerere University School of Medicine, Kampala, Uganda; 3 Department of Medicine, San Francisco General Hospital, University of California San Francisco, San Francisco, California, United States of America; 4 Division of Infectious Diseases, David Geffen School of Medicine, University of California Los Angeles, Los Angeles, California, United States of America; 5 Massachusetts General Hospital Center for Global Health and Harvard Medical School, Boston, Massachusetts, United States of America; University of Cape Town, South Africa

## Abstract

**Background:**

Late diagnosis of HIV infection is a major challenge to the scale-up of HIV prevention and treatment. In 2005 Uganda adopted provider-initiated HIV testing in the health care setting to ensure earlier HIV diagnosis and linkage to care. We provided HIV testing to patients at Mulago hospital in Uganda, and performed CD4 tests to assess disease stage at diagnosis.

**Methods:**

Patients who had never tested for HIV or tested negative over one year prior to recruitment were enrolled between May 2008 and March 2010. Participants who tested HIV positive had a blood draw for CD4. Late HIV diagnosis was defined as CD4≤250 cells/mm. Predictors of late HIV diagnosis were analyzed using multi-variable logistic regression.

**Results:**

Of 1966 participants, 616 (31.3%) were HIV infected; 47.6% of these (291) had CD4 counts ≤250. Overall, 66.7% (408) of the HIV infected participants had never received care in a medical clinic. Receiving care in a non-medical setting (home, traditional healer and drug stores) had a threefold increase in the odds of late diagnosis (OR = 3.2; 95%CI: 2.1–4.9) compared to receiving no health care.

**Conclusions:**

Late HIV diagnosis remains prevalent five years after introducing provider-initiated HIV testing in Uganda. Many individuals diagnosed with advanced HIV did not have prior exposure to medical clinics and could not have benefitted from the expansion of provider initiated HIV testing within health facilities. In addition to provider-initiated testing, approaches that reach individuals using non-hospital based encounters should be expanded to ensure early HIV diagnosis.

## Introduction

Early diagnosis of HIV infection is critical for improvement of HIV treatment outcomes [1]–[4]. Early diagnosis and treatment also reduces the cost of medical care [5]. Additionally, studies have suggested that early initiation of HIV treatment may have important prevention benefits [6]–[8]. As such, the recent World Health Organization (WHO) treatment guidelines recommend initiation of HIV treatment at CD4≤350, and several countries have adopted these new guidelines [9], [10]. However, treatment of all individuals with CD4≤350 will require earlier diagnosis of HIV infection.

There has been a drive to scale-up HIV Counseling and Testing (HCT) services in order to ensure early diagnosis and access to HIV services including care and treatment as well as prevention [11]. Increased access to HIV services is important for the attainment of the Millennium Development Goals for HIV as well as maternal and child health in sub-Saharan Africa [12]. Recent reports show improvement in access to HCT, yet over 60% of infected individuals globally remain unaware of their sero-status [11]. In 2005, it was estimated that 80% of HIV infected individuals in Uganda were unaware of their HIV status [13]. Research studies also reported late diagnosis and treatment of HIV infected individuals [14]. Late initiation of HIV treatment in sub-Saharan Africa has been associated with limited access to treatment but could also be attributed to delays in diagnosis of HIV infection and to delayed linkage to care after diagnosis [14], [15].

In an effort to scale-up access to HIV testing and linkage to care many countries have adopted new HCT approaches, including provider-initiated HIV testing and counseling (PITC) in the health care setting and home based HIV counseling and testing (HBHCT) [11], [16], [17]. In 2005, Uganda revised its HCT policy to include PITC and HBHCT [18]. The proportion of individuals who have tested and received HIV results in Uganda has increased over time; estimated at 10% in 2003, 23% in 2006, and 38% in 2008 [19]–[21]. In theory, increased access to HCT should lead to earlier diagnosis of HIV but this has not been evaluated. We provided HCT, determined prior HIV testing and medical history, and performed CD4 counts for patients newly diagnosed with HIV in the medical and emergency wards and the outpatient medical clinics in Mulago National Referral Hospital in Uganda and determined predictors of late HIV diagnosis.

## Methods

### Ethics Statement

The study was approved by Makerere University School of Medicine Ethics Committee, Uganda National Council for Science and Technology and the Institutional Review Boards of the University of California San Francisco and University of California Los Angeles. All study subjects provided written informed consent prior to participation.

### Study Setting

Mulago Hospital is the University teaching hospital of Makerere University School of Medicine, and is one of two national referral hospitals in Uganda. Mulago is the largest hospital in Uganda, serving over one million patients a year, and was the first hospital to initiate PITC in Uganda, in 2004 [22]. The medicine department in the hospital has five general medical wards (three non-private, one private, and one emergency ward) providing services for in-patients. When patients come to the hospital, they are screened in the emergency department. Adult patients are referred to the emergency medical ward for overnight care. Outpatient medical care is also provided within the medical outpatient clinic. Follow-up HIV care for adult patients diagnosed with HIV during hospitalization happens through several HIV clinics within the hospital, including the Infectious Disease Clinic (IDC) and the Mulago HIV clinic.

### Procedures

This study was conducted as part of an ongoing randomised, controlled trial to assess the impact of brief HIV counseling within PITC versus detailed counseling on linkage to HIV care and HIV risk reduction. Adult patients (≥18 years) who had never tested for HIV, or tested negative >1 year prior to recruitment, residing within 25 kms of Mulago hospital, willing to receive a HIV test, and possessing sufficient cognitive ability to participate in the study were eligible.

### Screening and enrollment

Following eligibility screening, written consent, baseline interviews and randomization, participants received HCT and CD4 testing immediately after HIV positive diagnosis. Between May 2008 and March 2010, 3613 patients were screened for eligibility and 1216 were ineligible. The most common reasons for being ineligible were testing for HIV within a year of screening (347), too ill to participate in the study (214), residence outside the 25 km radius (160), respondent planned to shift residence within a year of recruitment (157), and age <18 years (153). Of the 2,397 eligible participants, 1,998 (83.4%) agreed to participate in the study. Interview data was available for 1,966 participants at the time of analysis. Of the 616 who were HIV infected, 612 had CD4 cell counts done and were included in the analysis (Figure 1).

**Figure:**
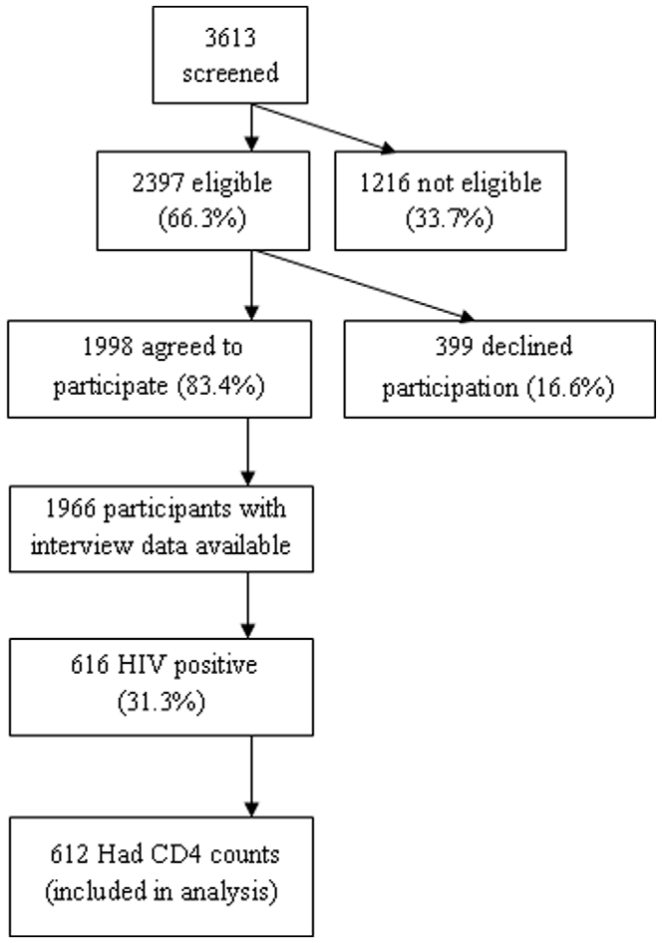
Screening and enrollment of study subjects. This figure shows the number of potential participants screened for eligibility, the number of eligible subjects who agreed to participate and received HIV testing, and the number of HIV infected participants who had CD4 tests.

### Laboratory testing

After the pre-test counseling, the counselor contacted the laboratory technician to draw blood for HIV testing. HIV testing was done using the serial testing algorithm with Determine (Abbott Laboratories, Abbott Park, IL), Uni-Gold (Trinity Biotech, Wicklow, Ireland) and Stat-Pak (Chembio Diagnostics). Blood for baseline CD4 cell counts for HIV positive participants was drawn immediately after disclosure of results. Quality control for HIV testing and CD4 tests were done at the Makerere University-Johns Hopkins University (MU-JHU) laboratory that is certified by the College of American Pathologists. All HIV infected individuals were provided with referrals to outpatient HIV clinics for follow-up care. Study participants received their CD4 test results and were advised to share the results with their providers.

### Measurements

Demographic characteristics, prior medical and HIV testing history, HIV risk perceptions and sexual risk behaviors were collected using interviewer administered structured interviews.

### Dependent variable

The primary dependent variable was late stage HIV at diagnosis, defined as CD4≤250, based on the current Ugandan guidelines for ART eligibility.

### Independent variables

#### Medical history and health seeking behavior

Participants were asked about experiences with medical services, ever and in the prior year, including hospital admissions, encounters with physicians, pharmacists, traditional healers, hospice, and other providers, and diagnoses of tuberculosis or genital herpes. We also created a summary variable to indicate whether or not the participant had ever received medical treatment in a “non-hospital setting” (from a pharmacy, home health care worker or traditional healer).

#### Risk perception and HIV risk behaviors

Participants were asked to describe the chances that they already had the HIV virus to assess risk perception. Participants were also asked about lifetime and recent (within 12 and 3 months of interview) sexual activity, including numbers of sexual partners, and with each of the five most recent sex partners in the prior 3 months, the relationship type, the frequency of condom use, whether they discussed HIV infection with this partner, and partner HIV status. Partner HIV status was categorized as known negative, known positive or unknown.

#### Anticipated outcomes of HIV diagnosis, disclosure, and participation in HCT

Participants were asked about expected positive and negative outcomes of being diagnosed with HIV infection, and plans for disclosure in the event of HIV positive sero-status. Potential positive outcomes of HIV testing and participation in HCT included improved care from doctors, access to HIV care and treatment, financial support, and increased support from family and sexual partner. Potential negative outcomes resulting from disclosure of HIV status included discrimination, breakup of relationships and physical abuse. We created a summary variable to indicate whether or not participants anticipated at least one of the eight negative outcomes of disclosure.

#### Other independent covariates

Socio-demographic variables included participant age, gender, education, occupation, religion, marital status, and income. Alcohol use was assessed by categorizing participants in three categories: those who never drank alcohol, past drinkers (those who last drank alcohol over one year ago), and current drinkers (those who had consumed alcohol within the past year). Heavy alcohol use was also assessed using the AUDIT-C, a three-item screening tool used to identify hazardous drinking.

### Analysis

As we were interested in late diagnosis of HIV, we limited all analyses to HIV-positive study participants with available CD4 counts (n = 612). The stage of diagnosis was first categorised as CD4≤250, >250–350, and >350. This was intended to capture the proportion of participants eligible for treatment at a CD4≤250 (current Uganda guidelines) as well as the cut-off of CD4≤350, as recommended by WHO. For all analyses, however, we compared participants with late stage HIV at diagnosis (CD4≤250, based on current Uganda guidelines), to those who did not present with late stage HIV. We conducted analyses using Chi-square tests to determine whether socio-demographic characteristics, reasons for testing, expected outcomes of testing, HIV risk behaviors or perceptions, or medical history were associated with late stage diagnosis. For variables that were also ordinal such as number of sexual partners or perceived risk of being infected with HIV, we additionally conducted the Armitage test for trend, excluding those who declined to answer and reported these trends when they were statistically significant. We used multivariate logistic regression to identify correlates of late stage diagnosis. All covariates that were associated with late presentation in the bivariate analyses at statistical significance level of p<0.20 were included in the preliminary multivariate model, with the exception of the following three variables: reasons for HIV testing, diagnosis of genital herpes within the past 5 years, and hospital admission within the past year. Genital herpes diagnosis was excluded because it was collinear with prior receipt of health care. In addition, lifetime and past year hospitalization were collinear and we included the former as a broad measure. We then reduced this multivariate model using backwards selection, forward selection, and stepwise selection (i.e. a combined backward and forward method), using a cut-off of p≤0.05 for inclusion/exclusion of variables in the models. All three selection models retained the same final explanatory variables as predictors of late stage HIV at diagnosis.

## Results

### Univariate analysis

The majority of participants were female (62.9%, 385), 18–30 years old (49.4%, 302), and of primary level education (53.9%, 330), and about half had a monthly income of <100,000 Uganda shillings (equivalent to <50 USD) (Table 1). Overall, 47.6% of participants (291) had CD4 counts ≤250, 11.6% (71) had CD4>250–350, and 40.9% (250) had CD4>350. The majority (489, 81.8%) had never tested for HIV. Among those who had previously tested for HIV, 65 (60.2%) tested>two years prior to the current test while 43 (39.8%) tested within one to two years of the current test.

**Table 1:** Participant social and demographic characteristics by stage of HIV at diagnosis.

Characteristic	Totaln (%)N = 612	CD4>250n (%)N = 321	CD4≤250n (%)N = 291	p-value
**Sex**				0.09
Male	227 (37.1)	109 (48.0)	118 (52.0)	
Female	385 (62.9)	212 (55.1)	173 (44.9)	
**Age** (median = 31; IQR = 26–38)				0.02
18–30 years	302 (49.4)	175 (58.0)	127 (42.1)	
31–45 years	262 (42.8)	121 (46.2)	141 (53.8)	
>45 years	48 (7.8)	25 (52.1)	23 (47.9)	
**Education**				0.64
No formal education	35 (5.7)	22 (62.9)	13 (37.1)	
Primary school	330 (53.9)	173 (52.4)	157 (47.6)	
Secondary school	213 (34.8)	111 (52.1)	102 (47.9)	
Primary or Ordinary Level professional	4 (0.7)	2 (50.0)	2 (50.0)	
Technical/University	30 (4.9)	13 (43.3)	17 (56.7)	
**Occupation**				0.80
Agriculture	32 (5.2)	16 (50.0)	16 (50.0)	
Business	269 (44.0)	136 (50.6)	133 (49.4)	
Other	252 (41.2)	138 (54.4)	114 (45.2)	
Unemployed	59 (9.6)	31 (52.5)	28 (47.5)	
**Monthly income (median = 60,000 USH; IQR = 10,000–150,000)**				0.41
<50,000	171 (27.9)	95 (55.6)	76 (44.4)	
50,000–100,000	97 (15.9)	49 (50.5)	48 (49.5)	
>100,000	134 (21.9)	65 (48.5)	69 (51.5)	
Don't know	205 (33.5)	111 (54.2)	94 (45.9)	
Declined	5 (0.8)	1 (20.0)	4 (80.0)	
**Religion**				0.86
Protestant	181 (29.6)	90 (49.7)	91 (50.3)	
Catholic	216 (35.3)	113 (52.3)	103 (47.7)	
Moslem	109 (17.8)	61 (56.0)	48 (44.0)	
Saved/Pentecostal	96 (15.7)	51 (53.1)	45 (46.9)	
Other	10 (1.6)	6 (60.0)	4 (40.0)	
**Marital status**				0.69
Married	251 (41.0)	127 (50.6)	124 (49.4)	
Married in the past	269 (44.0)	143 (53.2)	126 (46.8)	
Never married	92 (15.0)	51 (55.4)	41 (44.6)	
**Alcohol consumption history**				0.61
Never	220 (36.0)	117 (53.2)	103 (46.8)	
Past drinker (>1 year ago)	115 (18.8)	55 (47.8)	60 (52.2)	
Current drinker (within 1 year)	273 (44.7)	148 (54.2)	125 (45.8)	
Don't know	3 (0.5)	1 (33.3)	2 (66.7)	
**AUDIT-C alcohol risk group**				0.57
No alcohol	401 (68.3)	204 (50.9)	197 (49.1)	
Moderate risk	110 (18.7)	60 (54.6)	50 (45.5)	
Hazardous risk	76 (13.0)	43 (56.6)	33 (43.4)	

#### Medical history, health seeking behavior, and reasons for testing

Overall, 20.6% (126) of participants reported that they had ever received treatment in a medical clinic, 66.7% (408) had never received treatment in a medical clinic, while the rest were not sure/did not know if they had received treatment at a clinic. One hundred and twenty five (20.4%) had received treatment from a drug store or pharmacy while 7.8% (48) had received treatment from a traditional healer or herbalist. Only 70 participants (11.4%) reported having ever been admitted to a hospital in their lifetime; 30 (42.9%) of these reported being admitted within the past year. Twenty-seven (4.4%) reported a diagnosis of tuberculosis within 5 years, and 33.5% reported having ever been diagnosed with genital herpes. Wanting to know the HIV status and concerns about illness/AIDS symptoms were the main reasons for testing; 46.4% (284) and 46.2% (383), respectively (Table 2).

**Table 2:** Participant medical history and health seeking behavior by stage of HIV at diagnosis.

MEDICAL HISTORY AND HEALTH SEEKING BEHAVIOR	Total n (%)N = 612	CD4>250n (%) N = 321	CD4≤250n (%) N = 291	p-value
**Ever received treatment in a medical clinic**				<0.01
Yes	126 (20.6)	45 (35.7)	81 (64.3)	
No	408 (66.7)	228 (55.9)	180 (44.1)	
Don't know/Declined	78 (12.8)	48 (51.5)	30 (38.5)	
**Ever received treatment in a drug store/pharmacy**				<0.01
Yes	125 (20.4)	38 (30.4)	87 (69.6)	
No	406 (66.3)	233 (57.4)	173 (42.6)	
Don't know/Declined	81 (13.2)	50 (61.7)	31 (38.3)	
**Ever treated by a home health care worker**				<0.01
Yes	12 (2.0)	1 (8.3)	11 (91.7)	
No	522 (85.3)	272 (52.1)	250 (47.9)	
Declined	78 (12.8)	48 (61.5)	30 (38.5)	
**Ever treated by a traditional healer/herbalist**				<0.01
Yes	48 (7.8)	11 (22.9)	37 (77.1)	
No	486 (79.4)	262 (53.9)	224 (46.1)	
Declined	78 (12.8)	48 (61.5)	30 (38.5)	
**Ever received care from: pharmacy, home health care worker, or traditional healer**				<0.01
Yes	135 (22.1)	41 (30.4)	94 (69.6)	
No	399 (65.2)	232 (58.2)	167 (41.9)	
Declined	78 (12.8)	48 (61.5)	30 (38.5)	
**Ever admitted to the hospital**				<0.01
Yes	70 (11.4)	20 (28.6)	50 (71.4)	
No	459 (75.0)	251 (54.7)	208 (45.3)	
Don't know/Declined	83 (13.6)	50 (60.2)	33 (39.8)	
**Hospital admissions, past 12 months**				<0.01
0 admissions	497 (81.2)	266 (53.5)	231 (46.5)	
> = 1 admissions	30 (4.9)	5 (16.7)	25 (83.3)	
Don't know	7 (1.1)	2 (28.6)	5 (71.4)	
Declined	78 (12.8)	48 (61.5)	30 (38.5)	
**Tuberculosis diagnosis in past 5 years?**				0.21
Yes	27 (4.4)	10 (37.0)	17 (63.0)	
No	582 (95.1)	310 (53.3)	272 (46.7)	
Don't know/declined	3 (0.5)	1 (33.3)	2 (66.7)	
**Ever diagnosed with genital herpes**				0.09
Yes	205 (33.5)	96 (46.8)	109 (53.2)	
No	406 (66.3)	224 (55.2)	182 (44.8)	
**Recruitment site**				<0.01
Outpatient	366 (59.8)	225 (61.5)	141 (38.5)	
Medical Wards	149 (24.4)	51 (34.2)	98 (65.8)	
3BEM/CAS	97 (15.9)	45 (46.4)	52 (53.6)	
**Main reasons for wanting to test**				<0.01
AIDS symptoms/concerns about current illness	283 (46.2)	117 (41.3)	166 (58.7)	
Past sexual behaviors (participant's or partner's)	32 (5.2)	24 (75.0)	8 (25.0)	
Just wanted to know/plan for the future	284 (46.4)	174 (61.3)	110 (38.7)	
Other reasons/declined	13 (2.1)	6 (46.2)	7 (53.9)	

#### HIV Risk perception and sexual behavior

Over half (58%, 355) of the participants thought they could already be infected with HIV, 12.1% (74) thought they were probably already infected, and 10.6% (65) thought they were certainly already infected. Overall, 73.8% (451) reported sexual activity within 12 months; 29.7% (134) of these reported more than one sexual partner. Among those who were sexually active in the past 3 months (56.1%, 343), 58.3% (200) reported that their most recent partner was a spouse, and 50.4% (173) had discussed the risk of HIV infection with their most recent partner. The vast majority of those who were sexually active in the past 3 months had either not used condoms or had used them inconsistently (84.3%, 288); 14.9% (51) reported that their most recent sexual partner was HIV positive, 22.2% (76) reported that their most recent sexual partner was HIV negative, and 62.9% (215) reported that they did not know the HIV status of their most recent sexual partner (Table 3).

**Table 3:** Participant HIV risk perception and behaviors by stage of HIV at diagnosis.

RISK PERCEPTION AND RISK BEHAVIORS	Totaln (%)N = 612	CD4>250n (%)N = 321	CD4≤250n (%)N = 291	p-value
**Chances that you ** ***already have*** ** HIV virus**				0.20*
It almost certainly will not happen	53 (8.7)	28 (52.8)	25 (47.2)	
It could happen	355 (58.0)	199 (56.1)	156 (43.9)	
It probably will happen	74 (12.1)	35 (47.3)	39 (52.7)	
It almost certainly will happen	65 (10.6)	27 (41.5)	38 (58.5)	
Declined	65 (10.6)	32 (49.2)	33 (50.8)	
**Number of lifetime sexual partners (median = 4; IQR = 3–7)**				0.86
None	1 (0.2)	0 (0.0)	1 (100.0)	
1 partner	32 (5.2)	18 (56.3)	14 (43.8)	
2–5 partners	338 (55.3)	178 (52.7)	160 (47.3)	
>5 partners	157 (25.7)	82 (52.2)	75 (47.8)	
Don't know/Declined	83 (13.6)	43 (51.8)	40 (48.2)	
**Had sexual activity in 12 months**				0.46
Yes	451 (73.8)	241 (53.4)	210 (46.6)	
No	159 (26.0)	80 (50.3)	79 (49.7)	
Declined	1 (0.2)	0 (0.0)	1 (100.0)	
**Number of sexual partners, 12 months (median = 1; IQR = 0–1)**				0.45
None	166 (27.2)	83 (50.0)	83 (50.0)	
1 partner	303 (49.6)	154 (50.8)	149 (49.2)	
2–5 partners	128 (21.0)	76 (59.4)	52 (40.6)	
>5 partners	6 (1.0)	4 (66.7)	2 (33.3)	
Don't know/Declined	8 (1.3)	4 (50.0)	4 (50.0)	
**Number of sexual partners, 3 months (median = 1; IQR = 0–1)**				0.05
None	267 (43.8)	132 (49.4)	135 (50.6)	
1 partner	291 (47.7)	153 (52.6)	138 (47.4)	
2 partners	35 (5.7)	26 (74.3)	9 (25.7)	
> = 3 partners	17 (2.8)	10 (58.8)	7 (41.2)	
**Relationship with most recent sex partner (in 3 months)**				0.04
No partner	267 (43.8)	132 (49.4)	135 (50.6)	
Spouse	200 (32.8)	103 (51.5)	97 (48.5)	
Mistress/girl/boy friend/lover	115 (18.9)	74 (64.4)	41 (36.7)	
Other	28 (4.6)	12 (42.9)	16 (57.1)	
**Discussed risk of HIV infection with most recent partner**				0.09
Yes	173 (28.4)	103 (59.5)	70 (40.5)	
No	164 (26.9)	85 (51.8)	79 (48.2)	
No partner, past 3 months	267 (43.8)	132 (49.4)	135 (50.6)	
Don't know/Declined	5 (0.8)	1 (20.0)	4 (80.0)	
**Condom use, with most recent sex partner**				0.93
Always	54 (15.8)	29 (53.7)	25 (46.3)	
Sometimes	32 (9.4)	17 (53.1)	15 (46.9)	
Never	256 (74.9)	143 (55.9)	113 (44.1)	
**HIV status of most recent sex partner**				0.51
Positive	51 (14.9)	29 (56.9)	22 (43.1)	
Negative	76 (22.2)	46 (60.5)	30 (39.5)	
Don't know	215 (62.9)	114 (53.0)	101 (47.0)	

*p-value test for trend when declined group was excluded  =  0.04.

#### Anticipated outcomes of diagnosis with HIV infection

A large proportion of the participants expected positive outcomes of HIV diagnosis, disclosure, and participation in HCT. Participants were especially optimistic regarding access to HIV services; 90.3% (552) expected increased support from health workers, 98.7% reported that diagnosis of HIV would cause doctors to take better care of them, and 97.7% (597) expected to receive medications to treat HIV. A large proportion (77.6%, 474) also anticipated increased emotional support from their families and relatives upon disclosure of HIV positive status (Table 4).

**Table 4:** Anticipated positive outcomes of diagnosis by stage of HIV at diagnosis.

ANTICIPATED POSITIVE OUTCOMES	Totaln (%)N = 612	CD4>250n (%)N = 321	CD4≤250n (%)N = 291	p-value
Increased emotional support from family/relatives				0.73
Yes	474 (77.6)	248 (52.3)	226 (47.7)	
No	106 (17.4)	54 (50.9)	52 (49.1)	
Not applicable	12 (2.0)	8 (66.7)	4 (33.3)	
Declined	19 (3.1)	11 (57.9)	8 (42.1)	
Increased support from health professionals				0.58
Yes	552 (90.3)	294 (53.3)	258 (46.7)	
No	32 (5.2)	13 (40.6)	19 (59.4)	
Not applicable	2 (0.3)	1 (50.0)	1 (50.0)	
Declined	25 (4.1)	13 (52.0)	12 (48.0)	
**Positive outcomes of participating in HCT/HIV diagnosis**				
Doctor(s) take better care				0.76
Yes	603 (98.7)	317 (52.6)	286 (47.4)	
No	3 (0.5)	2 (66.7)	1 (33.3)	
Declined	5 (0.8)	2 (40.0)	3 (60.0)	
Receive medications to treat HIV				0.85
Yes	597 (97.7)	314 (52.6)	283 (47.4)	
No	7 (1.2)	4 (57.1)	3 (42.9)	
Declined	7 (1.2)	3 (42.9)	4 (57.1)	
Receive financial assistance from the government				0.48
Yes	146 (23.9)	82 (56.2)	64 (43.8)	
No	344 (56.3)	180 (52.3)	164 (47.7)	
Declined	121 (19.8)	59 (48.8)	62 (51.2)	
Receive financial assistance from other organizations				0.81
Yes	245 (40.1)	127 (51.8)	118 (48.2)	
No	237 (38.8)	123 (51.9)	114 (48.1)	
Declined	129 (21.1)	71 (55.0)	58 (45.0)	
Join an HIV/AIDS support group				0.46
Yes	525 (85.9)	281 (53.5)	244 (46.5)	
No	53 (8.7)	24 (45.3)	29 (54.7)	
Declined	33 (5.4)	16 (48.5)	17 (51.5)	
Continue to use other HIV/AIDS counseling services				0.16
Yes	577 (94.4)	308 (53.4)	269 (46.6)	
No	19 (3.1)	6 (31.6)	13 (68.4)	
Declined	15 (2.5)	7 (46.7)	8 (53.3)	
Use hospice services				0.03
Yes	452 (74.0)	247 (54.7)	205 (45.4)	
No	117 (19.2)	49 (41.9)	68 (58.1)	
Declined	42 (6.9)	25 (59.5)	17 (40.5)	
Receive treatment from a traditional healer or herbalist				0.62
Yes	80 (13.1)	46 (57.5)	34 (42.5)	
No	507 (83.0)	262 (51.7)	245 (48.3)	
Declined	24 (3.9)	13 (54.2)	11 (45.8)	

Despite the high numbers of expected positive outcomes, a significant proportion of participants also anticipated negative outcomes of being diagnosed with HIV; 46.4% (281) anticipated at least one of eight negative outcomes, which included: breakup of marriage (9.7%, 59), physical abuse by the spouse (13.9%, 85), being neglected by their families (12.8%, 78), discrimination by employers (6.4%, 39), and estranged by peers (14.1%, 86); Table 5.

**Table 5:** Anticipated negative outcomes of diagnosis by stage of HIV at diagnosis.

ANTICIPATED NEGATIVE OUTCOMES	Totaln (%)N = 612	CD4>250n (%)N = 321	CD4≤250n (%)N = 291	p-value
**Anticipates at least one negative outcome of disclosing HIV+ status**				0.47
Yes	281 (46.4)	144 (51.3)	137 (48.8)	
No	325 (53.6)	176 (54.2)	149 (45.9)	
**Negative outcomes of disclosing HIV+ status**				
Breakup of marriage				0.05
Yes	59 (9.7)	25 (42.4)	34 (57.6)	
No	167 (27.3)	90 (53.9)	77 (46.1)	
Not applicable	344 (56.3)	191 (55.5)	153 (44.5)	
Declined	41 (6.7)	15 (36.6)	26 (63.4)	
Physical abuse by spouse/sexual partner				0.83
Yes	85 (13.9)	42 (49.4)	43 (50.6)	
No	267 (43.7)	142 (53.2)	125 (46.8)	
Not applicable	223 (36.5)	116 (52.0)	107 (48.0)	
Declined	36 (5.9)	21 (58.3)	15 (41.7)	
Neglected by family				0.76
Yes	78 (12.8)	44 (56.4)	34 (43.6)	
No	491 (80.4)	257 (52.3)	234 (47.7)	
Not applicable	9 (1.5)	5 (55.6)	4 (44.4)	
Declined	33 (5.4)	15 (45.5)	18 (54.6)	
Disowned by family				0.79
Yes	42 (6.9)	21 (50.0)	21 (50.0)	
No	547 (89.5)	288 (52.7)	259 (47.4)	
Not applicable	9 (1.5)	6 (66.7)	3 (33.3)	
Declined	13 (2.1)	6 (46.2)	7 (53.9)	
Discrimination by health professionals				0.53
Yes	27 (4.4)	16 (59.3)	11 (40.7)	
No	531 (86.9)	276 (52.0)	255 (48.0)	
Not applicable	4 (0.7)	1 (25.0)	3 (75.0)	
Declined	49 (8.0)	28 (57.1)	21 (42.9)	
Breakup of sexual relationships				0.29
Yes	150 (24.6)	76 (50.7)	74 (49.3)	
No	213 (34.9)	122 (57.3)	91 (42.7)	
Not applicable	194 (31.8)	99 (51.0)	95 (49.0)	
Declined	54 (8.8)	24 (44.4)	30 (55.6)	
Discrimination by employers				0.35
Yes	39 (6.4)	19 (48.7)	20 (51.3)	
No	236 (38.6)	116 (49.2)	120 (50.9)	
Not applicable	318 (52.5)	178 (56.0)	140 (44.0)	
Declined	18 (3.0)	8 (44.4)	10 (55.6)	
Estranged by peers				0.63
Yes	86 (14.1)	41 (47.7)	45 (52.3)	
No	449 (73.5)	237 (52.8)	212 (47.2)	
Not applicable	26 (4.3)	16 (61.5)	10 (38.5)	
Declined	50 (8.2)	27 (54.0)	23 (46.0)	

#### Comparisons between early and late diagnosis

Among participants with late stage HIV infection, 48.5% (141) were 31–45 years old, compared to 37.7% (121) among those with CD4>250 (p = 0.02). There were no other significant socio-demographic differences between the two groups (Table 1). Of the participants who reported having ever received treatment in a non-hospital setting (home, traditional healer and drug stores), 41 (30.4%) had late stage HIV compared to 232 (58.2%) among those who had not received such care (p<0.01) (Table 2). Among the reasons for testing for HIV, concerns about ill health were more prominent among individuals with late diagnosis (p<0.01). The site of participant recruitment was associated with late stage HIV diagnosis; 65.8% (98) of the participants who were recruited from the medical wards had CD4≤250 while only 38.5% (141) of those who were recruited from the outpatient clinics had CD4≤250 (p<0.01). When we conducted a test for trend among those who did not decline the question, there was an inverse relationship between belief that one was infected with HIV and CD4 cell count at testing (Table 3). There was borderline statistical evidence that more of the participants who presented late, compared to those who did not present late, anticipated that disclosing HIV+ status would break up a marriage (p = 0.05) (Table 5).

### Multivariate predictors of late diagnosis

We conducted forward, backward, and stepwise selection and all three methods resulted in the same final model. In the final multivariate model, only three variables: age, receipt of non-medical health care, and the number of recent sexual partners were the only variables independently associated with late presentation. Participants who were 31–45 years old had 1.6 times the odds of being diagnosed late compared to participants who were 18–30 years old (95% CI: 1.1–2.3); Table 6. Participants who reported ever receiving care in a non-medical setting (home, traditional healer and drug stores) also had significantly increased odds of late stage diagnosis, compared to those who had never received care from such providers (OR = 3.2; 95%CI: 2.1–4.9). Compared to participants with no sexual partners in the past three months, those reporting two partners had decreased odds of late diagnosis (OR = 0.3; 95%CI: 0.1–0.7). Participants with three or more partners also had decreased odds of late diagnosis, although this association was not statistically significant (p = 0.3).

**Table 6:** Multivariate predictors of late stage HIV at diagnosis (CD4≤250).

Characteristic	Odds Ratio	95% Confidence Interval
Age		
18–30 years	1.00	—
31–45 years	1.63	(1.15, 2.30)
>45 years	1.00	(0.52, 1.92)
Ever received health care from a pharmacy, home health worker, or a traditional healer		
Never	1.00	—
Ever	3.20	(2.09, 4.91)
Declined	0.86	(0.52, 1.44)
Number of sex partners, past three months		
0 partners	1.00	—
1 partner	0.87	(0.62, 1.24)
2 partners	0.30	(0.13, 0.70)
3 or more partners	0.59	(0.20, 1.73)

## Discussion

We found that half of the newly diagnosed HIV infected patients at the national referral hospital in Uganda had late stage HIV infection. Upon multivariate analysis, older age and ever having received health care from a non-medical provider were associated with higher odds of late presentation, while being sexually active was associated with earlier presentation. Individuals within the 31–45 year age group were more likely to be diagnosed late possibly because they had been infected earlier. Similarly, those who had received care from a non-medical provider were more likely to be diagnosed late because the illness for which they sought care could have been HIV related, or because they did not go to medical facilities that offered HIV testing. Individuals who reported two sexual partners were less likely to have late HIV infection compared to those who reported no partners, possibly because such persons may also have experienced previous sexually transmitted infections and therefore had more contact with health facilities.

A significant proportion of those with late stage HIV (27.8%) had received care at a medical clinic and a similar proportion (29.9%) had previously received treatment from a pharmacy/drug store. Fewer individuals had prior encounters with traditional healers (7.8%) or home health workers (2.0%). These findings highlight several gaps and missed opportunities for HIV diagnosis, including: 1) Failure to diagnose HIV infection for infected individuals who attended medical clinics; and 2) A missed opportunity to make a diagnosis for HIV (or any other illness) for those who received care at the pharmacies and drug shops, since these outlets only sell drugs largely on request from sick individuals. Strengthening and expansion of PITC to all health units may reduce missed opportunities for HIV diagnosis, for those individuals who do make contact with these facilities. The initial scale-up of PITC focused more on the high HIV prevalence facilities, including medical and tuberculosis wards [22]. We found that two thirds of the participants in the medical wards had late stage HIV compared to one third of the outpatients. In comparison to the medical wards, HIV testing in outpatient units may provide a better opportunity for earlier HIV diagnosis [23].

Notably, two thirds of the newly diagnosed HIV infected individuals had never attended a medical clinic at all and could therefore not have benefitted from the expansion of provider initiated HIV testing within health facilities. Late stage diagnosis was also associated with receiving care from a non-medical facility. This indicates that a significant proportion of HIV infected individuals may not make contact with health facilities until late in their illness. Facility based interventions may not reach out to all individuals that require HCT because those who do not have health problems or are not ill will not go to health facilities. Also, some individuals with health problems may opt to seek care elsewhere. An estimated 13% of Ugandans who need and seek healthcare do so from drugstores [24]. The scale-up of PITC also initially targeted public facilities. Yet, only 29% of Ugandans seek healthcare at public facilities while 46% go to private clinics [24]. Scale-up of PITC and other facility based interventions should involve the private sector. Augmentation of PITC with community and home based HIV testing approaches will also be critical in expanding access to early diagnosis and care. A study that compared four HCT approaches in Uganda found that the HCT approaches were complementary. While a larger proportion of infected individuals was identified through PITC, HBHCT identified HIV infected individuals at an earlier stage of infection [25]. Additionally, providers at pharmacies, drug stores, and traditional healers should encourage people to seek additional services including HIV testing at medical facilities. Strategies to improve early diagnosis will be critical to the current efforts of early initiation of HIV treatment – most of the participants in this study (59%) had CD4 counts of <350.

Several previous studies showed that low perception of risk of being infected with HIV may be associated with failure to utilise HIV testing services [26]. We found an inverse relationship between risk perception and CD4 count on univariate analysis, suggesting that many do not feel that they are at risk until they become ill. However, risk perception was not statistically significant in the multivariate models, possibly due to an association with seeking medical care. In addition to the suspicion that they could be HIV infected, over 95% of the participants in this study noted the medical benefits of HIV diagnosis, including improved care and access to HIV treatment. Many also expected increased support from their families if they were to test positive. Although the proportion of participants mentioning each of the negative social outcomes of HIV disclosure was quite small, ranging between 6%–14%, about 46% anticipated at least one negative social outcome. It is possible that these negative social issues may have been a deterrent to testing [26], [27]. In a related study conducted in the same hospital, we found that the frequency of negative social outcomes was actually quite low [28]. However, these fears, real or exaggerated, need to be addressed through efforts to minimize stigma and discrimination, so that they do not hinder uptake of HIV services.

These data show significant HIV risk behavior. Most of the newly diagnosed HIV infected individuals were still sexually active and had partners who were either known HIV negative or of unknown HIV status. Yet, consistent condom use was very low. Diagnosis of HIV infection may lead to increased condom use since studies have shown less risky behavior including increased condom use following HIV diagnosis [29]. Better still, initiation of HIV treatment may reduce the risk of HIV transmission to sexual partners [8], [9], [29].

While we had a high response rate, our sample of individuals at a national referral hospital in an urban setting may not represent the larger Ugandan population. Also, these data were derived from a study which was not primarily designed to assess reasons for delayed diagnosis, and do not fully address some potential reasons for delayed diagnosis, including access to HIV testing. We believe that the lack of strong associations may be due to the fact that many of the participants in this study came to the hospital primarily to seek medical care rather than HIV testing. It is possible that the predictors of late diagnosis would be different if individuals who seek HIV testing late were compared with those who seek testing early. However, the study highlights issues that are critical to the mix of HIV testing approaches for the scale-up of early HIV diagnosis and linkage to HIV prevention and treatment.

In summary, this study highlights the need to strengthen PITC, and to augment the current PITC scale-up with community or other non-health facility based HCT approaches, as well as providing an environment that minimises the negative social outcomes of HIV diagnosis and disclosure of HIV status.
